# Fostering interactive medical education: integrating Web 2.0 tools through a universal design for learning framework

**DOI:** 10.1080/10872981.2026.2639193

**Published:** 2026-02-27

**Authors:** Esin Ergonul, Sibel Buyukcoban

**Affiliations:** a Dokuz Eylul University, Izmir, Turkey; b Anesthesiology and Reanimation, Dokuz Eylul University, Faculty of Medicine, Izmir, Turkey

**Keywords:** Teaching technologies, teaching methods, instructional tools, medical education, universal design for learning, web 2.0

## Abstract

**Background:**

The effective use of technology is essential for fostering inclusive and engaging learning environments.

**Objective:**

This study evaluated the impact of an experiential faculty development program based on Universal Design for Learning (UDL) principles and the use of Web 2.0 tools. We examined how this program influenced the integration of technology among medical educators.

**Design:**

A total of 262 faculty members participated from 2020 to 2022. Two months post-training, data were collected via surveys and focus groups.

**Results:**

Results showed a significant increase in participants' use of previously unfamiliar tools, particularly Google Forms (50%) and Wooclap (29.5%). Participants cited pedagogical reasons (such as ease of use, increased student engagement, and support for inclusivity) as key drivers of adoption. In contrast, time constraints and lack of technical support were key barriers to broader use.

**Conclusions:**

This study underscores the value of hands-on, practice-based training in digital pedagogy and highlights the importance of institutional support to sustain meaningful and widespread technology integration in medical education.

## Introduction

Achieving effective and lasting learning outcomes requires a fundamental shift from traditional, instructor-centric approaches to more interactive, student-centred educational designs [1–3]. While strong interaction among students, instructors, and educational tools is critical, conventional faculty development programmes often address it through didactic methods and fail to provide a robust, evidence-based framework for pedagogical practice [4–6].

The UDL model has emerged as a framework to meet this need, especially by creating flexible and accessible learning environments that accommodate diverse student needs [7]. Drawing from cognitive neuroscience [8].; Inc, n.d.; [9], UDL offers a systematic and evidence-based approach to designing learning materials, instructional methods, and assessment processes [10,11]. UDL is intended to assist students in answering three fundamental questions: ‘Why am I learning this?’, ‘What will I learn?’, and ‘How will I use this new knowledge?’ Each of these questions activates different areas of the brain, thereby making learning more physiologically and biochemically effective and accessible [8,9].

The effective integration of technology is a cornerstone of UDL. As Karl Luke emphasises, ‘appropriately utilise technology and digitise resources, presenting content in alternative formats (text, audio, and/or visual)’ [12]. Web 2.0 tools represent a transformative class of technologies that can fundamentally reshape teaching and learning. Unlike static resources, these tools foster dynamic student engagement through interactive videos, real-time Q&A platforms, and collaborative mind mapping. While the potential of Web 2.0 tools in medical education is recognised, a meta-analysis in 2016 found that they had not been sufficiently utilised [13]. More critically, a significant gap exists in the literature regarding the practical, evidence-based integration of UDL principles with Web 2.0 tools within medical education [14,15].

However, fully realising the potential of UDL and Web 2.0 tools is not only a technical task. It also depends on pedagogical, personal, and institutional factors. The literature indicates that educators may exhibit ‘resistance’ to technology adoption, and this resistance often stems not from superficial reluctance but from deeper structural barriers. Many educators also report technostress when technologies change quickly and when guidance is limited. For this reason, successful integration needs both adequate infrastructure (e.g., access and costs) and institutional readiness, including attention to social concerns such as the digital divide, data security, and ethical issues [16,17].

Medical education is a high-pressure field where heavy workloads and perfectionism-related stress can lead to overreliance on technology. In these instances, educators might treat technology as a primary dependency instead of a pedagogical aid. Such reliance can cause skill atrophy, including reduced critical thinking. It may also cause ‘pedagogical erosion’ by diminishing educator-student interaction and instructional creativity. Consequently, technology integration requires evidence-based frameworks that improve digital literacy and self-efficacy and address these specific pedagogical risks [17,18].

To address this critical gap, our study presents a novel two-year investigation into a faculty development programme designed to bridge pedagogy and technology. This study aims not only to evaluate faculty members' perceptions and adoption of UDL-aligned Web 2.0 tools but also to provide a deeper qualitative understanding of their experiences. By utilising a mixed-methods design that combines quantitative survey data with in-depth qualitative data from interviews and focus groups, this research moves beyond simple self-reported outcomes to provide a rich, nuanced analysis of the pedagogical impacts of this integrated approach. We believe this manuscript offers a valuable contribution to the fields of technology-enhanced education and inclusive pedagogy, providing an important model for improving interactive medical education.

## Materials & methods

This study utilised a mixed-methods research design to provide a comprehensive analysis of medical educators' experiences. This approach combined quantitative data from a survey with qualitative data from semi-structured interviews and focus groups to gain a deeper understanding of the pedagogical impact of integrating UDL principles and Web 2.0 tools. This study adhered to the Declaration of Helsinki and was approved by the Dokuz Eylul University Health Sciences Scientific Research and Publication Ethics Committee (Decision No: 3, Date: April 3, 2023). All participants took part voluntarily in the study, and informed consent to participate was obtained from all individuals.

Between 2020 and 2022, 262 faculty members participated in a faculty development programme conducted remotely, designed according to the UDL framework. Training sessions were held via the university’s distance education platform, using PowerPoint presentations and Web 2.0 tools. The programme aimed to enable faculty members to gain practical experience integrating these technologies into flexible and interactive educational environments consistent with UDL principles. The specific Web 2.0 tools were selected based on their accessibility, ease of use, interactivity, and their capacity to align effectively with UDL principles (Tables 1 and 2). Each four-hour training session was repeated 20 times over two years to ensure broad faculty participation. Interactive videos, collaborative group activities, and real-time Q&A sessions were included in each training session.

**Table 1.** Web 2.0 tools and their features.

Web 2.0 Tool	Description
Padlet	An online visual collaboration platform for creating and sharing digital boards. Supports diverse content types, ideal for brainstorming, discussion, and collaborative projects. [https://padlet.com]
Wooclap	Enhances student engagement with real-time quizzes, polls, and interactive activities, integrated with presentation tools and learning management systems. [https://www.wooclap.com]
Thinglink	An interactive media platform allowing creation of engaging content by adding hotspots to images, videos, and 360-degree panoramas. [https://www.thinglink.com]
Mindomo	An online mind mapping tool supporting collaborative visual idea mapping with multiple export formats and integrations. [https://www.mindomo.com]
Mindmeister	Real-time collaborative online mind mapping software for visualising and sharing ideas, tasks, and projects. [https://www.mindmeister.com]
Edpuzzle	An interactive video learning platform allowing educators to add questions, audio, and notes to videos with analytics on student progress. [https://edpuzzle.com]
Google Forms	Customisable survey tool integrated with Google Drive for creating and analysing forms, quizzes, and polls. [https://www.google.com/forms/about/]
Formative	Real-time assessment tool providing feedback and analytics on student performance, supporting diverse question types. [https://www.goformative.com]
Kahoot	Game-based learning platform promoting student engagement, collaboration, and real-time assessments through interactive quizzes. [https://kahoot.com]

**Table 2.** UDL Principles, brain regions involved, and Web 2.0 tools used during training.

UDL Principle [7]	Dominant Brain Region	Questions Addressed to Participants	Web 2.0 Tools Used
Engagement‘Why am I learning this?’	Limbic system	- I am attending this training because…- What would I most like to improve in live online teaching? - KWL (Know, Want to Know, Learned) activity related to auditory anatomy	Wooclap, Padlet
Representation‘What am I going to learn?’	Occipital area	- Which interactive learning techniques can be applied in the classroom? - How can I present information effectively using visual and auditory tools? - Concept mapping for a hypertension lesson- Interactive educational video about infectious diseases- Skill assessment for medical secretarial tasks (appointment scheduling)	Thinglink, Mindmeister, Mindomo, Edpuzzle
Action & Expression‘How will I use what I learned?’	Frontal area	- What am I taking away from this training? (Exit Ticket)- Practical demonstrations on platforms- Feedback on educational content and Web 2.0 tools	Wooclap, Formative, Kahoot, Google Forms

(Mindmeister,[19]; Padlet,[20]; Mindmodo,[21]; Edpuzzle,[22]; Wooclap,[23]; Thinglink,[24]; Formative,[25]; Kahoot,[26]; Google,[27]).

### Data collection and analysis

Quantitative data were collected using semi-structured surveys at two time points: before and two months after the training. The survey inquired about participants' demographic characteristics, the frequency of their use of Web 2.0 tools, and their opinions and suggestions. The frequency of use was measured on a 5-point scale. Additionally, participants’ satisfaction with the training was evaluated at the end of each session using a 5-point Likert scale. Quantitative data were analysed using descriptive statistics; continuous variables were presented as median (min–max), standard deviation, and standard error of the mean; categorical variables were presented as frequency and percentage.

To analyse changes in the adoption of UDL principles (use vs. non-use) before and after the training, the McNemar test was employed.

To examine differences in the frequency of Web 2.0 tool use (1 = never; 5 = very frequently) before and after the training, the nonparametric Wilcoxon signed-rank test was applied.

Effect sizes (r) were calculated for significant changes using the formula r = Z/√ *N* to determine the practical significance of the results. The statistical significance level was set at *p* < 0.05 for all tests.

To gain a deeper, more nuanced understanding of the educators’ perspectives, qualitative data were collected through semi-structured interviews and focus group discussions. Fifteen participants were selected via purposive sampling for individual interviews, each lasting approximately 30 minutes. Additionally, two focus groups, each with five participants, were conducted to explore shared experiences and collective insights. We used interviews and focus groups to explore how educators experienced tool use in practice, including what helped, what was difficult, and how classroom participation was affected. Data were analysed using inductive thematic analysis [28], enriched by an interpretive phenomenological lens [16].

## Results

This section presents a comprehensive, mixed-methods analysis of medical faculty members’ approaches to adopting Web 2.0 tools and implementing UDL principles. The findings integrate quantitative survey data, which provide an overview of tool adoption, with rich qualitative data from interviews and focus groups, which offer deeper insight into the educators' pedagogical motivations and shifts.

### Quantitative findings: adoption and perceived utility

The demographic and professional characteristics of the participants are summarised in Table 3. The majority were female (57.3%) and were aged 30–49 years (64.9%).

**Table 3.** Demographic and professional characteristics of the participants.

Item		*n* (%)
Gender	Male	112 (42.7)
	Female	150 (57.3)
Age	20–29	9 (3.4)
	30–39	89 (34.0)
	40–49	81 (30.9)
	50–59	52 (19.8)
	60-69	31 (11.8)
The years of teaching experience	1–4 years	46 (17.6)
	5–9 years	54 (20.6)
	10–14 years	51 (19.5)
	15-19 years	63 (24.0)
	20 and + years	48 (18.3)
Discipline	Basic Sciences Departments	86 (32.8)
	Clinical Sciences Departments	89 (34.0)
	Surgical Sciences Departments	87 (33.2)

Overall satisfaction with the training programme was moderately high (M = 3.90, SD = 1.1), with participants giving the highest scores to the ‘Pace of the Training’ and ‘Likelihood of Recommending the Training’ (Table 4).

**Table 4.** Satisfaction with the components of the training programme (%).

Evaluation Category	1	2	3	4	5	Mean (SD/SEM)
Overall satisfaction with the training	1.9	7.6	28.2	22.5	39.7	3.90 (1.1/0.07)
Adequacy of training preparation	–	–	34.7	35.5	29.8	3.95 (0.80/0.05)
Appropriateness of the training method	–	–	42.4	28.2	29.4	3.87 (0.83/0.05)
Sufficiency of training duration	–	24.8	29.0	25.6	20.6	3.41 (1.07/0.07)
Appropriateness of the training pace	–	–	32.4	32.4	35.1	4.03 (0.82/0.05)
Likelihood of recommending the training	1.9	7.6	28.2	22.5	39.7	3.90 (0.82/0.05)

Likert scale: 1=Not satisfied at all; 2=Not satisfied; 3=Neutral; 4=Satisfied; 5=Very satisfied.

The training led to a significant increase in the application of UDL principles. While no participants reported using UDL principles before the training, their post-training application of UDL principles showed a notable increase, particularly for the Engagement (39.7%) and Representation (35.9%) principles. The analysis demonstrated a statistically significant increase in the adoption of all three UDL principles (Table 5).

**Table 5.** McNamer’s test results for pre and post training adoption of UDL principles (*N* = 262).

UDL Principles	Pre/post ‘yes’ n	McNamer ki-square	p
Enhancing student engagement (Engagement)	0/104	104	<0.001
Presenting information in multiple ways (Representation)	0/94	94	<0.001
Supporting multiple means of expression (Action & Expression)	0/78	78	<0.001

Similarly, the use of Web 2.0 tools shifted significantly. While tools like Wooclap, Padlet, and Edpuzzle were virtually unused before the training, a marked increase in their adoption was observed afterward. Notably, Google Forms and Wooclap exhibited high post-training adoption rates, with 50% and 29.5% of participants, respectively, reporting frequent or very frequent use. As shown in Table 6, the analysis confirmed a statistically significant increase in the frequency of use of the tools covered in the training. The effect sizes indicated large effects for these increases (Google Forms r = 0.77, Padlet r = 0.64, Wooclap r = 0.62).

**Table 6.** Pre- and post-training distribution of participants' usage of Web 2.0 tools.

Web 2.0 Aracı	Pre Mean(SD/SEM)	Post Mean (SD/SEM)	Z	p
Whatsapp	4.88 (0.48/0.03)	5.00 (.00/0.00)	–4.000	<.001
Google Forms	2.18 (0.98/0.06)	3.15 (1.57/0.09)	–12.448	<.001
Padlet	1.00 (.00/0.00)	2.81 (1.92/0.13)	–10.392	<.001
Wooclap	1.00 (.00/0.00)	2.28 (1.59/0.09)	–10.030	<.001
Blogs	1.48 (1.08/0.07)	1.31 (0.67/0.04)	–4.262	<.001
Edpuzzle	1.00 (.00/0.00)	1.76 (1.08/0.07)	–10.481	<.001
Mindmeister	1.00 (.00/0.00)	1.53 (0.65/0.04)	–10.657	<.001
Mindmodo	1.00 (.00/0.00)	1.53 (0.64/0.04)	–10.796	<.001
Formative	1.09 (0.42/0.03)	1.46 (0.70/0.04)	–9.291	<.001
Kahoot	1.02 (0.17/0.01)	1.29 (0.61/0.04)	–7.438	<.001
Thinglink	1.00 (.00/0.00)	1.10 (0.50/0.03)	–2.877	.004
Smartboard	1.00 (.00/0.00)	1.00 (.00/0.00)	.00(.00/0.00)	1.00

Wilcoxon Signed Test; SEM: Standard error mean, SD: Standard deviation.

1 = Never; 2 = Rarely (less than once per month); 3 = Sometimes (1 to 2 times per month); 4=Frequently (3 to 5 times per month); 4 = Very frequently (6 or more times per month).

#### Qualitative findings: pedagogical approaches and practical perceptions

Thematic analysis revealed that the use of Web 2.0 tools was not merely a technical adaptation process, but rather a complex experience intertwined with power dynamics, emotional labour, and elements of institutional resistance.

Theme 1: Shifting Power Dynamics and Pedagogical Vulnerability: Participants’ statements suggested a tendency to move away from the traditional ‘knowledge-transmitting authority’ role by incorporating tools like Wooclap. One participant’s remark, ‘I will use these to understand what students know and do not know,’ might indicate an intention to shape the lesson flow based on students’ immediate needs and, therefore, a willingness to share control. However, this process may have triggered a sense of ‘pedagogical vulnerability’ in some participants. Specifically, concerns such as ‘I need to be ready for the answers provided’ or ‘discussing the results might extend the lesson duration’ could be interpreted as both a fear of losing absolute dominance and anxiety about managing unplanned discussions. Furthermore, anxieties expressed regarding ‘technical problems’ may not only reflect concerns about physical disruptions but also fears of appearing vulnerable before the class and of losing authority.

Theme 2: Inclusion as Emotional Labour: Platforms such as Wooclap and Padlet, which facilitate anonymous participation, were perceived not merely as technical tools but potentially as vital sources of ‘emotional safety’ for students. One participant’s observation, ‘This will help students who are hesitant to speak,’ may exemplify the invisible efforts educators expend in managing the classroom’s emotional climate to ensure students’ sense of security. Furthermore, remarks such as ‘*I worry about the silent students and want to give them a voice without putting them on the spot’* suggest that the drive for inclusivity can be an emotionally demanding process. This indicates that educators may perform a form of emotional labour as they constantly seek to balance student anxiety with pedagogical objectives.

Theme 3: A Reaction to Institutional Constraints: Resistance and Technostress: Thematic analysis suggests that participants’ remarks regarding ‘*preparation processes requiring significant time and effort*’ may reflect not only practical challenges but also a form of ‘resistance’ in response to a lack of institutional support. Statements such as ‘It is not easy for me to use’ can be linked to the concept of ‘technostress’ as described by [17]. In this context, the frequent expression ‘I don’t have time’ may not solely indicate a lack of time, but may also represent a coping strategy to safeguard their professional space and academic confidence within the high-pressure environment of medical education. This suggests that what appears to be a logistical barrier may, in fact, be a protective response to institutional pressures.

**Figure 1.**
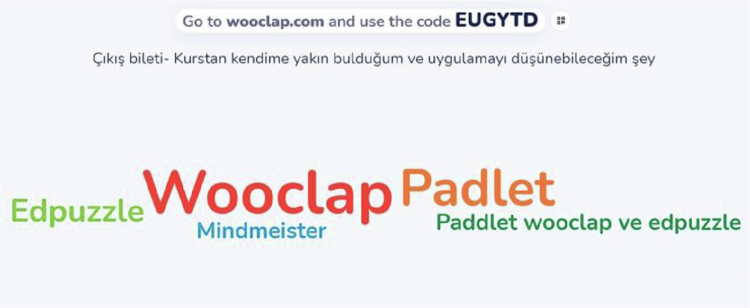
The results of the ‘Exit Ticket’ activity were collected on the Wooclap.

Thematic analysis provided in-depth insights into the educators' approaches. The word cloud generated by the ‘Exit Ticket’ activity shows that tools such as Wooclap and Padlet were the most memorable and the most likely to be implemented by participants (Figure 1).

#### Pedagogical practice: aligning Web 2.0 tools with UDL domains

In this section, participants’ tool-usage practices were examined with reference to the guideline principles established by CAST (Centre for Applied Special Technology) [29] and the Web 2.0 principle alignments outlined in the methodology (Table 2) to concretise the qualitative themes within the UDL framework:

- Action & Expression (Guidelines 4 and 5): The data confirmed that using Wooclap and Google Forms addressed the goal of Physical Action (Guideline 4). These tools diversified modes of interaction by allowing participants to respond via mobile devices rather than by raising their hands. In addition, within the context of Expression and Communication (Guideline 5), offering digital channels as alternatives to verbal speech enabled educators to overcome their pedagogical vulnerability (silent classrooms) and to shift students from passive to active roles.
- Engagement (Guideline 7): The use of Padlet, which was associated with activation of the limbic system in the study design (Table 2), aligned directly with CAST’s principle of Recruiting Interest. Its capacity to support anonymous participation emerged as a key feature in minimising threats. This enabled educators to manage the emotional labour of inclusivity (Theme 2) and to foster the participation of reserved students in a low-risk environment.
- Representation (Guideline 1): Although adoption rates were lower due to time constraints (Theme 3), tools such as Edpuzzle and Mindomo, which are linked to the occipital region and the visual presentation in Table 2, proved to be critical resources for UDL’s perception principle. These tools diversified access to information by enabling delivery through customisable visual and auditory channels rather than text alone.

## Discussion

The most significant finding of this study is that although medical teachers were introduced to UDL for the first time, approximately one-third of them began to implement UDL designs following the training. UDL has been described, particularly since the 1990s, as a framework aiming to enhance students’ active participation in the learning process [8,30]. A study conducted in Ireland and the United Kingdom reported that only 31% of anatomy educators were aware of the UDL concept [11]. The finding that none of the participants had prior familiarity with UDL is consistent with studies reporting low levels of awareness of UDL. After the training, the shift toward an ‘initial implementation’ level suggests a positive short-term response. However, given the two-month follow-up period and the use of self-reported measures, no generalisation can be made regarding the sustainability or long-term transformative impact of these results, and therefore no definitive conclusion can be drawn.

This faculty development programme led to a statistically significant increase in both the adoption of UDL principles and the use of Web 2.0 tools. The relatively high post-training uptake of Google Forms (50%) and Wooclap (29.5%) indicates an initial interest in student engagement. However, most educators did not use these tools after the training, suggesting that the programme’s impact remained at an early stage rather than being broad and sustained. This pattern is consistent with prior studies examining barriers to technology adoption [16–18].

The contextual limitations identified in the qualitative findings (such as ‘lack of time’ and ‘heavy workload’ under the theme of overcoming practical barriers) reflect broader systemic issues frequently highlighted in the technology integration literature [17,18]. Recent studies emphasise that the primary obstacles to technology integration include infrastructure deficiencies and high costs; a lack of clear institutional policy and vision; and, particularly, the heterogeneity of educators’ digital competencies [17,18]. In this context, although the programme demonstrated success, the adoption rate of a pedagogically valuable tool such as Wooclap remained low (29.5%), which is noteworthy. This suggests that overcoming institutional and individual challenges to UDL-based technology integration requires more than a one-time successful intervention; it necessitates continuous and structured support.

The second key finding of the study concerns the frequency of Web 2.0 tool usage. Before the training, participants rarely or never used any tools other than WhatsApp. However, after the training, the proportion of participants who frequently used Google Forms increased to 50%, and those who used Wooclap rose to 29.5%. This increase can be partially explained by the Technology Acceptance Model (TAM), which posits that perceived ease of use and perceived usefulness drive technology adoption [31,32]. Our qualitative findings support this, as participants emphasised the ‘easy-to-learn interface’ and ‘interactive features’ of these two platforms, indicators of perceived usefulness, which are consistent with previous literature [33–35]. Additionally, this is consistent with more recent models suggesting that the hedonic motivation (i.e., enjoyment) associated with technology also enhances adoption [36].

However, viewing these adoption rates solely through an optimistic lens would be incomplete. While 50% of participants did not adopt Google Forms, the non-adoption rate was even higher for Wooclap, at 70.5%. These figures point to significant barriers that cannot be fully explained by the TAM’s perceived usefulness construct alone. The literature on technology integration identifies several potential barriers: individual differences (such as a natural inclination to try new tools, digital literacy, academic confidence, and levels of self-efficacy) [17,18,36]; institutional factors (such as workload and time constraints) [18]; and technostress [16,17]. Therefore, the 29.5% adoption rate likely does not reflect low perceived usefulness of the tool, but rather indicates that educators lacked the opportunity to implement this innovation because of institutional and individual barriers, such as workload and technostress.

Despite these challenges, participants recognised the potential of Web 2.0 tools to support UDL’s ‘Action & Expression’ principle. This principle is often considered difficult to implement in practice. The adoption of tools such as Wooclap and Google Forms indicates educators' willingness to offer digital alternatives to traditional methods, such as hand-raising. These findings suggest that participants view digitalisation as more than just moving materials to a screen. Instead, they see it as a genuine opportunity to change classroom interaction. By using these channels, educators gained experience managing pedagogical vulnerability. Furthermore, they demonstrated a trend toward the more democratic and student-centred model of authority described by [16]. In addition to the partial adoption challenges observed with Google Forms and Wooclap, the negligible usage rates of other tools, such as Mindomo and Edpuzzle, confirm that difficulties adapting to new technologies remain a significant barrier. This reinforces a claim in the literature [37] that effective implementation of digital tools requires institutional support and sharing best practices. The ‘technical problems’ and ‘lack of familiarity with technology’ reported by participants in the qualitative findings align with the concepts of ‘insufficient digital infrastructure,’ ‘low digital literacy,’ and ‘low academic self-efficacy’ described in the current literature.

The deep-belief change emphasised by [38] appears to have been initiated through the adoption of UDL principles in this study; however, the translation of this change into practice was limited by systemic barriers, as the qualitative findings also revealed [38]. In particular, medical educators' resistance to technology, due to a ‘heavy workload’ [18], and technostress resulting from a lack of institutional support [17] hinder the adoption of more complex tools. In this context, the word cloud generated from the ‘Exit Ticket’ activity (Figure 1) visually confirms that user-friendly tools such as Wooclap and Padlet were the preferred choices. This suggests that educators, by gravitating toward pedagogically valuable yet low-friction tools, aimed to minimise the risk of technostress described by [17] while developing strategies to safeguard their professional competence [17,18].

### Limitations and strengths

Among the limitations of this study is that the use of Web 2.0 tools was measured only two months after the training, which may not capture long-term usage trends or sustainability. Longer follow-up periods are needed for a more comprehensive assessment. Additionally, because the data are based on participants’ self-reports, there is the potential for response bias. Direct observation of in-class implementations or examination of student feedback could offer deeper insights into the effectiveness of digital tools. Lastly, as this research was conducted within a specific institutional context, caution should be exercised in generalising the findings.

On the other hand, the study’s strengths include the combined use of quantitative and qualitative data, enabling a holistic and reliable analysis of the results. The participation of 262 medical teachers from various fields also supports the broader applicability of the findings within medical education. Moreover, collecting data at two different time points (before and after the training) provides a longitudinal perspective on the adoption of Web 2.0 tools. Focusing on the use of technology within the UDL framework further underscores the importance of inclusive and student-centred educational strategies.

## Conclusions

This study demonstrates that a faculty development programme grounded in UDL principles and integrated with Web 2.0 tools can enhance the digital pedagogical skills of medical educators in the short term. While the results show a significant increase in adoption, the effectiveness of the programme should be interpreted within the constraints of the two-month evaluation window. Rather than claiming a transformative impact, these findings offer a promising model for early-stage technology integration that requires further longitudinal validation.

The integration of UDL with user-friendly Web 2.0 tools provides a robust model for creating more interactive, inclusive, and student-centred learning environments in medical education. However, to ensure the sustainability and scalability of these benefits, a broader institutional commitment is essential. Future success will depend on providing sustained professional development, dedicated technical support, and the systematic sharing of best practices. This study offers a clear pathway for institutions aiming to optimise digital pedagogy and enhance interactive learning experiences for diverse student populations.

During the preparation of this manuscript, the authors used ChatGPT to improve the academic English writing style. After utilising this tool, the authors thoroughly reviewed and edited the content as necessary and took full responsibility for the final version of the publication.

## Data Availability

The datasets used or analysed during the current study are available from the corresponding author upon reasonable request.
